# Cutaneous Stigmata of Occult Spinal Dysraphism

**Published:** 2013-01-01

**Authors:** Yogesh Kumar Sarin

**Affiliations:** Department of Pediatric Surgery, Maulana Azad Medical College, New Delhi.

A boy was born with a patch of hair over the spine with a dimple at its cranial aspect (Fig. 1). The pediatrician assured the parents that the hair would eventually fall out and the dimple was harmless. Later on, parents visited another physician for the toddler's constipation and urinary tract infections, who recognized significance of hairy patch at sacrum. The magnetic resonance imaging (MRI) was advised which showed syringomyelia and spinal cord tethering. 

**Figure:**
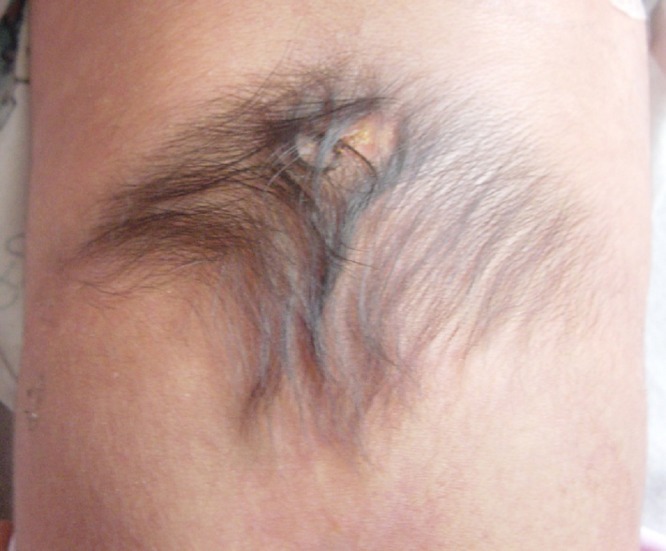
Figure 1: Note hypertrichiosis (Faun tail) with a patch of aplasia cutis congenital and dermal sinus tract.

A girl was born with unusual marks along the midline of the lower back, a lipoma with a dimple and a hemangioma (Fig. 2). The obstetrician reassured the parents, nothing to worry about. The baby was referred for an MRI to a tertiary center, on advice of pediatrician. The baby was ultimately diagnosed to have lipomyelomeningocele.

**Figure:**
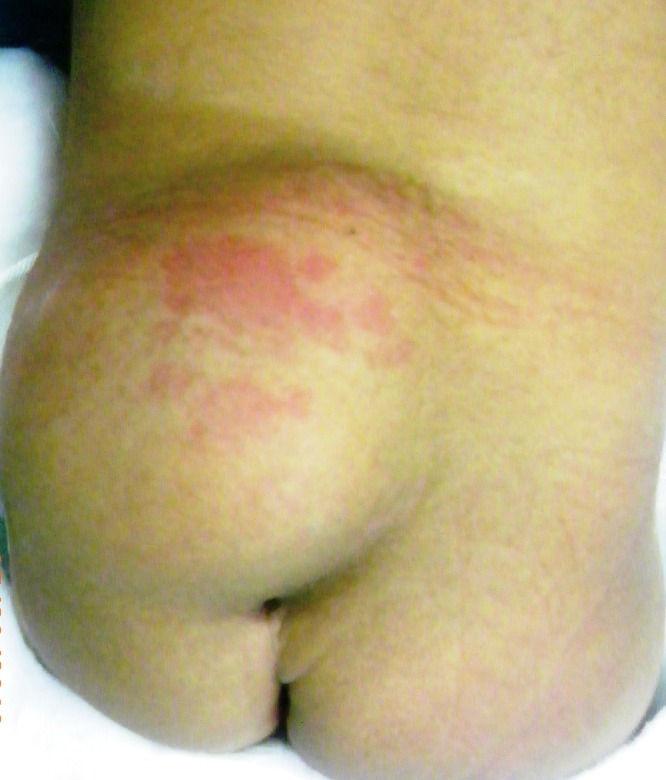
Figure 2: Note lipoma, cutaneous hemangioma, and atypical dimple.

Occult spinal dysraphism (OSD) is congenital absence of a spinous process and variable amounts of lamina with no visible exposure of meninges or neural tissue. OSD encompasses many entities such as lipomeningomyelocele, tethered spinal cord, dermal sinus tracts with or without dermoid/ epidermoids, diastematomyelia, meningocoele manqué, neurenteric cysts, terminal myelocystocele. Without early surgery, these children might suffer irreversible neurological deficits and are at high risk of recurrent meningitis, bladder and bowel incontinence, and disabling motor deficits [1]. 

The various cutaneous markers for OSD include; midline or paraspinal mass, capillary hemangioma (Port-wine stain), hairy patch, dermal sinus, polypoid lesions or rudimentary tail or caudal appendage, atretic meningocele, or "cigarette burn" sign, Nevi or hyper pigmentation, aplasia cutis congenita, dyschromic lesions (hypopigmented macule, hyperpigmented macule, cafe´ au lait macule and mongolian spot), and deviated natal cleft. These signs might occur anywhere along the midline but are seen most frequently in the lumbar region. A combination of 2 or more congenital midline skin lesions is more reliable marker of OSD [1]. Some cutaneous marker even when present alone, such as lipoma, tail, and dermal sinus tracts would warrant imaging studies [1]. On the other hand ‘isolated’ cutaneous hemangioma, hypertrichiosis, pigmented nevus, Mongolian spot may have a very low incidence of underlying OSD and may not merit further investigation in absence of any symptoms [1]. 

A spinal ultrasound (US) in first 6 months of life is often sufficient for confirming the OSD [2, 3], but ultrasonography is operator dependent. Some lesions, such as a dermal sinus tract going into the lower end of the spinal cord, may be missed even by an experienced radiologist. In an Indian multi-centric study, US in infants of less than 6 months and in cases of flat cutaneous stigmata missed only 5% of OSD, but in cases with bulky overlying masses (lipoma, hemangioma), it missed 15% of cases [4]. A spinal MRI is the most sensitive diagnostic imaging technique [1], but is expensive and usually needs anesthesia.

## Footnotes

**Source of Support:** Nil

**Conflict of Interest:** The author belongs to the editorial team, however the manuscript is dealt independently by other editors and the author did not participate in decision making of the manuscript.
